# Transcriptomics and metabolomics analysis reveal the dietary copper deficiency and supplementation effects of liver gene expression and metabolite change in grazing sheep

**DOI:** 10.1186/s12864-024-10134-3

**Published:** 2024-02-27

**Authors:** Xiwei Jin, Lingbo Meng, Zhi Qi, Lan Mi

**Affiliations:** School of Life Sciences, Inner Mongolia University, Hohhot, 010000 China

**Keywords:** Copper, Deficiency, Metabolomics, Sheep, Supplementation, Transcriptomics

## Abstract

**Background:**

The appropriate mineral nutrients are essential for sheep growth and reproduction. However, traditional grazing sheep often experience mineral nutrient deficiencies, especially copper (Cu), due to inadequate mineral nutrients from natural pastures.

**Results:**

The results indicated that dietary Cu deficiency and supplementation significantly reduced and elevated liver concentration of Cu, respectively (*p* < 0.05). *FOXO3*, *PLIN1*, *ACTN2*, and *GHRHR* were identified as critical genes using the weighted gene co-expression network analysis (WGCNA), quantitative real-time polymerase chain reaction (qRT-PCR), and receiver operating characteristic curve (ROC) validation as potential biomarkers for evaluating Cu status in grazing sheep. Combining these critical genes with gene functional enrichment analysis, it was observed that dietary Cu deficiency may impair liver regeneration and compromise ribosomal function. Conversely, dietary Cu supplementation may enhance ribosomal function, promote lipid accumulation, and stimulate growth and metabolism in grazing sheep. Metabolomics analysis indicated that dietary Cu deficiency significantly decreased the abundance of metabolites such as cholic acid (*p* < 0.05). On the other hand, dietary Cu supplementation significantly increased the abundance of metabolites such as palmitic acid (*p* < 0.05). Integrative analysis of the transcriptome and metabolome revealed that dietary Cu deficiency may reduce liver lipid metabolism while Cu supplementation may elevate it in grazing sheep.

**Conclusions:**

The Cu content in diets may have an impact on hepatic lipid metabolism in grazing sheep. These findings provide new insights into the consequences of dietary Cu deficiency and supplementation on sheep liver and can provide valuable guidance for herders to rationalize the use of mineral supplements.

**Supplementary Information:**

The online version contains supplementary material available at 10.1186/s12864-024-10134-3.

## Background

Sheep, as an essential livestock species, play a vital role in contributing significantly to humanity across various realms, including food production, textile manufacturing, and agriculture [1]. The Inner Mongolia Autonomous Region holds a significant position in the livestock sector of China. The livestock economy is regarded as one of the crucial industries in the region and serves as the primary economic backbone for the local herders [2]. Nonetheless, several studies have shown that grazing sheep may not receive sufficient mineral intake, such as copper (Cu), from natural forages to support their optimal growth and development [3–5]. Cu deficiency is one of the most frequent mineral element deficiencies in grazing ruminants [6].

Cu is a micromineral element required for animal growth and reproduction. It is a key component of a large number of enzymes, such as superoxide dismutase, cytochrome C oxidase, and caeruloplasmin. These are associated with iron (Fe) homeostasis, Cu transport, angiogenesis, immune function, and blood cell maturation [6, 7]. Cu deficiency in grazing ruminants has been reported in many articles, such as Tibetan antelope in the Qinghai Lake Basin of China, sheep in the Qilian Mountain Plateau of China, and cattle calves in the Salado River Basin of Argentina [4–6]. Cu deficiency occurs more frequently in ruminants than in monogastric mammals because redox reactions in the rumen lead to thiomolybdate formation from sulfide and molybdate, which in turn generates insoluble material with Cu in digestive juices, thus significantly reducing the efficiency of Cu uptake by ruminants [7]. Inadequate intake of Cu will lead to reproductive disorders and reduced fertility in animals, as well as reduced concentration of norepinephrine in the brains of rats and sheep [6, 8]. Further research has indicated that an extended deficiency of Cu in sheep can greatly elevate their mortality rate [9]. In the presence of Cu deficiency, it also leads to the down-regulation of genes involved in mitochondrial and peroxisomal fatty acid β-oxidation in mice [10]. Thus far, the impact of Cu deficiency and supplementation on gene expression and metabolite changes in the liver is still not fully understood.

Transcriptomic and metabolomic are useful tools for exploring gene and metabolite change in animals. Therefore, this experiment was conducted with a total of 28 Inner Mongolian grazing Wu Ranke sheep, which were evenly divided into a control group and Cu treatment group. The Cu treatment group was fed with Cu deficient multi-nutrient salts for 60 days and then fed with Cu supplement multi-nutrient salts for 41 days. The liver fresh weight, concentration of essential mineral elements, transcriptomic, and metabolomic analyses of 28 grazing Mongolian sheep were detected to explore the effects of Cu deficiency and supplementation on liver gene expression and metabolite change in grazing sheep. The aim of this study was to gain novel insights into the impacts of Cu deficiency and supplementation on the liver of grazing sheep. The findings can serve as a valuable guide for herders regarding the rational use of mineral supplements to enhance the overall well-being of grazing sheep.

## Methods

### Animal ethics

All animal-related procedures were conducted in strict adherence to the guidelines and regulations set forth by the Inner Mongolia University Animal Care and Use Committee (IMU-2020-sheep-040).

### Experimental design

Twenty-eight 4-month-old female grazing Wu Ranke sheep were procured from Abaga Banner in the Xilin Gol League of the Inner Mongolia Autonomous Region. These sheep were individually housed and provided a diet consisting of 400 g crushed oat, 1000 g natural forage, and multi-nutrient salts as recommended by the National Research Council (NRC, 2007) [11]. The composition and dosage of multi-nutrient salts were provided in Table S1, while the nutrient content of oat and natural forage can be found in Table S2.

**Fig. 1:**
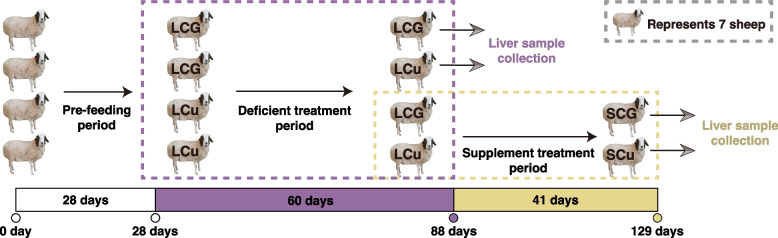
Feeding experimental design of the copper treatment group and control group in 28 grazing Wu Ranke sheep. Abbreviations: LCu = the low-Cu feeding group; LCG = the control group for the low-Cu feeding period; SCu = the high-Cu supplement group; SCG = the control group for the high-Cu feeding period

Throughout the 28-day pre-feeding period, the sheep were exclusively provided a diet consisting of crushed oats and native pasture. At the end of the pre-feeding period, 28 Wu Ranke sheep were randomly divided into the low-Cu feeding group (LCu) and the control group for the low-Cu feeding period (LCG). LCu and LCG were respectively fed with Cu deficient multi-nutrient salts and standard multi-nutrient salts for 60 days. Then, 7 sheep were randomly selected from each group for slaughter, while the remaining 7 sheep in each group continued fed with Cu supplement multi-nutrient salts and standard multi-nutrient salts. After 41 days of feeding, these sheep were also slaughtered, serving as the high-Cu supplement group (SCu) and the control group for the high-Cu feeding period (SCG) (Fig. 1).

### Sample collection

The Wu Ranke sheep were fasted for 12 h before slaughter. The liver samples were collected at the end of the Cu deficient treatment period (day 88) and the end of the Cu supplement treatment period (day 129) of the feeding period. The liver samples were weighed the fresh weight, then snap-frozen in liquid nitrogen and stored at -80 °C for later laboratory analysis. Throughout the entire experimental process, we employed disposable tubes devoid of RNA-degrading ribonucleases to guarantee the absence of any contamination caused by these enzymes.

### Detection of essential mineral elements in liver

The fresh liver samples were digested by a microwave digester (REVO, Labtech, Beijing, China). The digestion procedure of the microwave digester was shown in Table S3 [12].

The concentration of 10 minerals was measured using a TXRF spectrometer (S4 T-STAR, Bruker Nano GmbH, Berlin, Germany) equipped with a molybdenum (Mo) X-ray tube. The elements analyzed included phosphorus (P), sulfur (S), potassium (K), calcium (Ca), manganese (Mn), iron (Fe), cobalt (Co), Cu, zinc (Zn), and selenium (Se). A voltage of 50 kV and a current of 1000 uA for a duration of 300 s per sample for measurement. All the results of liver mineral elements measurement were calibrated with the standard curve [11].

### Transcriptomic analysis

#### RNA extraction and Illumina sequencing

Total RNA was isolated and purified from each liver sample using TRIzol reagent (Ambion, TX, USA) according to the manufacturer’s instructions. The RNA Nano 6000 Assay Kit of the Bioanalyzer 2100 system (Agilent Technologies, CA, USA) was used to assess the RNA integrity. Five sheep livers in each group were randomly selected to construct cDNA libraries using the NEBNext® Ultra™ RNA Library Prep Kit for Illumina®. The cDNA libraries from 20 Wu Ranke sheep livers for sequencing using the Illumina Novaseq platform and 150 bp paired-end reads were generated [13].

#### Quality control, reads mapping to the reference genome and quantification of gene expression level

The quality of the raw data was initially evaluated by FastQC software (https://www.bioinformatics.babraham.ac.uk/projects/fastqc), and the results included the base composition information of the sequence and the corresponding sequence quality information.

Download the sheep (Oar_rambouillet_v1.0) reference genome and gene annotation files from the Ensembl website (http://www.ensembl.org/index.html). Build the reference genome index by Salmon software and the reads were separately aligned to the reference genome [14]. Gene expression of each transcript was calculated and expressed as transcripts per kilobase million reads (TPM) [15].

#### Identification of differentially expressed genes

Differentially expressed genes (DEGs) between LCu and LCG, SCu and SCG were identified by the DESeq2 R package. The significant DEGs were determined based on | log_2_foldchange|≥ 1 and false discovery rate (FDR) < 0.05 (the *p*-value adjusted by the Benjamini–Hochberg method) [16].

#### Weighted gene co-expression network analysis

The weighted gene co-expression network analysis (WGCNA) is a bioinformatic analysis method that can be used to efficiently explore the relationship between genes and phenotypic information. The DESeq2 normalized gene expression data were used to construct co-expression networks for LCu, SCu, LCG and SCG by the WGCNA R package. The highly co-expressed gene modules were inferred using WGCNA for 9265 genes in LCu, SCu, LCG and SCG Wu Ranke sheep. PickSoftThreshold was used to select and verify an optimum soft threshold. In order to find modules based on topological overlap, the matrix data were transformed into an adjacency matrix, and then clustered. Clustering dendrograms were generated after the computation of module eigengene (ME) and the merging of related modules in the tree based on ME. This experiment used the concentration of Cu in the liver as the phenotypic information to screen for the hub gene associated with phenotypic information [17].

#### Screening of the critical genes

The genes identical to DEGs and hub genes were identified as candidate critical genes and then the receiver operating characteristic curve (ROC) model was verified for the selected critical genes using the pROC R package [17].

#### Quantitative real-time polymerase chain reaction validation

Gene expression levels were determined using quantitative real-time polymerase chain reaction (qRT-PCR). The Reverse Transcription Kit was used to reverse-transcribe total RNA into cDNA (R222, Vazyme, Nanjing, China). Using the SYBR Green master mix performed qRT-PCR as follows: one cycle at 95°C for 30 s, 40 cycles at 95°C for 10 s and 60°C for 30 s (Q311, Vazyme, Nanjing, China). The final volume was 10 uL (containing 1 uL of cDNA, 5 uL of SYBR Green mix, 0.2 uL each of both forward and reverse primers and 3.6 uL ddH_2_O). Real-time detection of SYBR Green fluorescence was conducted using a qTOWER 2.2 Real-Time PCR System (Analytik Jena, Jena, German). The GAPDH gene was amplified to serve as an internal control. The relative quantification values for critical genes were calculated by the 2^−ΔΔCt^ method [18]. The gene-specific primers were designed using Primer-BLAST (https://www.ncbi.nlm.nih.gov/tools/primer-blast/) (listed in Table S4).

#### Functional enrichment analysis

The ClusterProfiler R package was used to perform Gene Ontology (GO) enrichment of DEGs.

Gene Set Enrichment Analysis (GSEA) was a computational approach to determine if a pre-defined Gene Set can show a significant consistent difference between two biological states. The genes were ranked according to the degree of differential expression in the two samples, and then the predefined Gene Set was tested to see if they were enriched at the top or bottom of the list. Gene set enrichment analysis can include subtle expression changes. In this experiment, the GESA software (v 4.1.0) was used to GO enrichment data set for performing GESA enrichment.

The GO and GSEA enrichments with the FDR less than 0.05 and 0.25 were significant, respectively [17, 19].

### Metabolite extraction and untargeted metabolomic analysis

A total of 28 LCu, SCu, LCG, and SCG Wu Ranke sheep liver samples were extracted metabolites by standard procedures [20]. The sample extracts were analyzed by a Vanquish UHPLC system coupled with an Orbitrap Q ExactiveTM HF mass spectrometer (ThermoFisher, MA, USA). The chromatographic and mass spectrometric conditions are shown in Table S5.

The raw data files generated by UHPLC-MS/MS were processed using the Compound Discoverer 3.1 (CD3.1, ThermoFisher) to perform peak alignment, peak picking, and quantitation for each metabolite. After that, peak intensities were normalized to the total spectral intensity. The normalized data were used to predict the molecular formula based on additive ions, molecular ion peaks and fragment ions. And then peaks were matched with the mzCloud, mzVault and MassList databases to obtain accurate qualitative and relative quantitative results.

Variable importance in projection (VIP) ≥ 1, |log_2_ fold change|≥ 1 and *p* < 0.05 were identified as the significantly accumulated metabolites (DEMs) between LCu and LCG, SCu and SCG. The functions of the DEMs enriched pathways were studied using the Kyoto Encyclopedia of Genes and Genomes (KEGG) database (https://www.genome.jp/kegg/pathway.html). When the metabolic pathway *p* value was less than 0.05, the metabolic pathway was considered statistically significant enrichment [18, 21].

### Statistical analysis

The Shapiro–Wilk test was performed to assess the normality of data distribution. The independent samples t-test was performed to determine the significant difference between liver weight and liver mineral element concentration using GraphPad Prism (v.9.3.1). The results were reported as mean ± standard error (SE), and statistical significance was determined when the *p*-value was less than 0.05. Pearson’s correlation analysis was conducted between critical genes and DEMs using GraphPad Prism (v.9.3.1). The *p*-value of less than 0.05 and the correlation coefficient (r) absolute value of more than 0.7 were identified as significant correlations. The data visualization was accomplished using R (v.4.1.2), while Adobe Illustrator 2020 was employed for vector drawing.

## Results

### Fresh weight of the liver

**Fig. 2:**
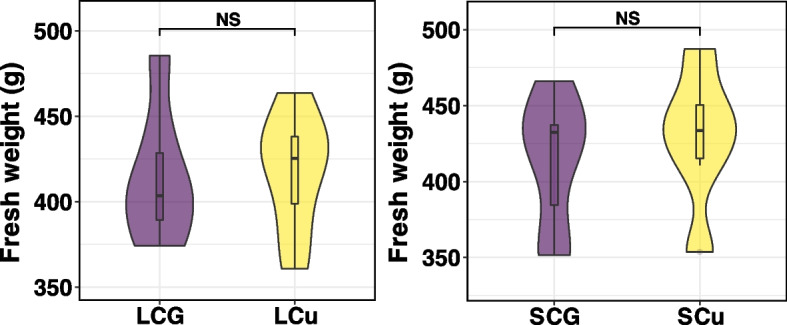
Fresh weight of grazing sheep liver. NS *p* > 0.05, * *p* < 0.05, ** *p* < 0.01, *** *p* < 0.001. Abbreviations: LCu = the low-Cu feeding group; LCG = the control group for the low-Cu feeding period; SCu = the high-Cu supplement group; SCG = the control group for the high-Cu feeding period

As shown in Fig. 2, the deficiency and supplementation of Cu in the diets did not affect the liver weight of Wu Ranke sheep (*p* > 0.05).

### Concentration of liver mineral elements

**Fig. 3:**
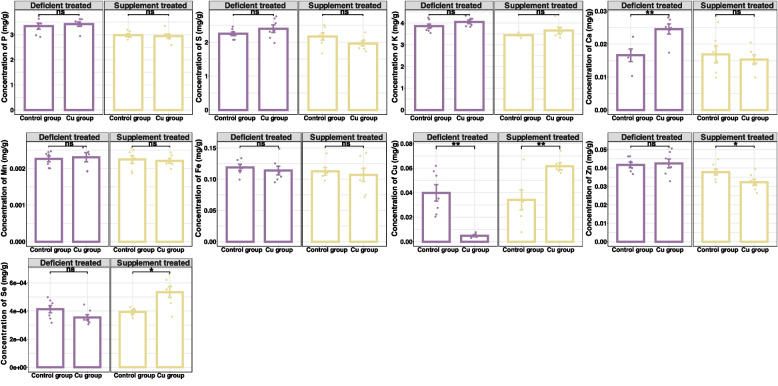
The concentration of liver mineral elements in copper deficient treated and supplement treated compared with control group. NS *p* > 0.05, * *p* < 0.05, ** *p* < 0.01, *** *p* < 0.001

Figure 3 showed the concentration of liver mineral elements in the Cu deficient treated and the Cu supplement treated.

After Cu deficient treatment, the liver concentration of Cu (0.0049 ± 0.00087 mg/g v.s. 0.040 ± 0.0066 mg/g, *p* < 0.05) was reduced sharply and the concentration of Ca (0.025 ± 0.0016 mg/g v.s. 0.017 ± 0.0019 mg/g, *p* < 0.05) increased significantly compared with LCG. There were no significant differences observed in the liver concentration of P, S, K, Mn, Fe, Zn and Se in the LCu compared with LCG. The concentration of Co below the detection limit of the instrument was not detected.

Following the Cu supplement treatment significantly increased the liver concentration of Cu (0.062 ± 0.0027 mg/g v.s. 0.034 ± 0.0082 mg/g, *p* < 0.05) and Se (0.00053 ± 0.000039 mg/g v.s. 0.00039 ± 0.000015 mg/g, *p* < 0.05) but reduced the liver concentration of Zn (0.032 ± 0.0017 mg/g v.s. 0.038 ± 0.0016 mg/g, *p* < 0.05) compared with the SCG. Cu supplement treatment had no effect on the liver concentration of P, S, K, Ca, Mn and Fe. The concentration of Co below the detection limit of the instrument was not detected.

### Transcriptome analysis

#### DESeq2 identification of DEGs

**Fig. 4:**
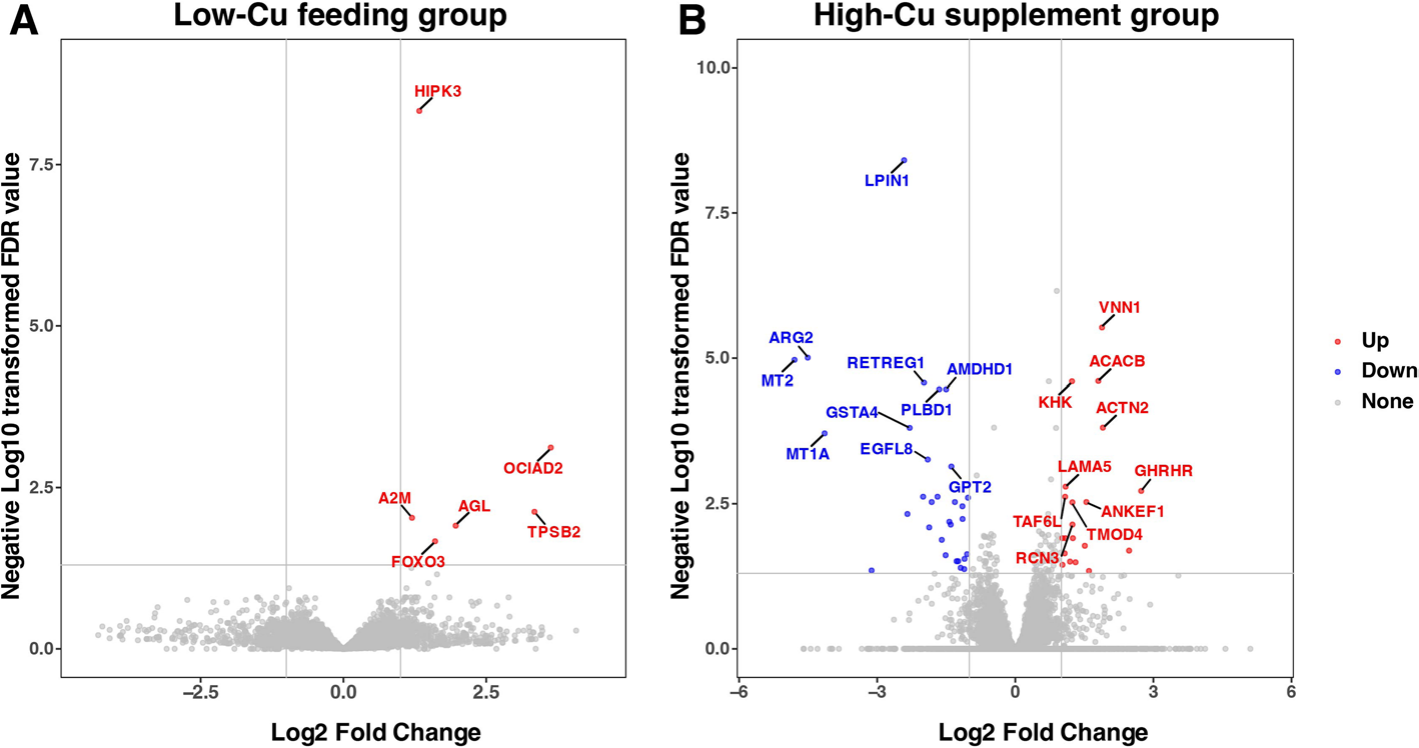
DEGs identified between different treatment groups. **A** Volcano Plot of DEGs in the copper deficient group. **B** Volcano Plot of DEGs in the copper supplement group. The top 10 up-regulated and down-regulated gene names were tagged and sorted by FDR value. Abbreviations: DEGs = differentially expressed genes; FDR = false discovery rate

As shown in Figure S1, the sequence quality and per sequence quality scores of LCu, LCG, SCu and SCG indicated a high quality of RNA-seq data. In order to find DEGs, gene expression was normalized using DESeq2, the normalized data are shown in Figure S2. A total of 6 up-regulated DEGs were identified between LCu and LCG (Fig. 4A), while 50 DEGs were identified between SCu and SCG, including 20 up-regulated and 30 down-regulated DEGs (FDR < 0.05) (Fig. 4B). The DEGs up-regulation and down-regulation genes information were shown in Table S6.

#### Functional enrichment analysis of DEGs

The GO enrichment results showed that DEGs of LCu were significantly enriched in 10 terms, such as oocyte maturation, positive regulation of regulatory T cell differentiation, DNA damage response, signal transduction by p53 class mediator, endopeptidase inhibitor activity, inclusion body, glycogen biosynthetic process, hydrolase activity, acting on glycosyl bonds, brain morphogenesis, neuronal stem cell population maintenance, and extrinsic apoptotic signaling pathway in absence of ligand (FDR < 0.05).

**Fig. 5:**
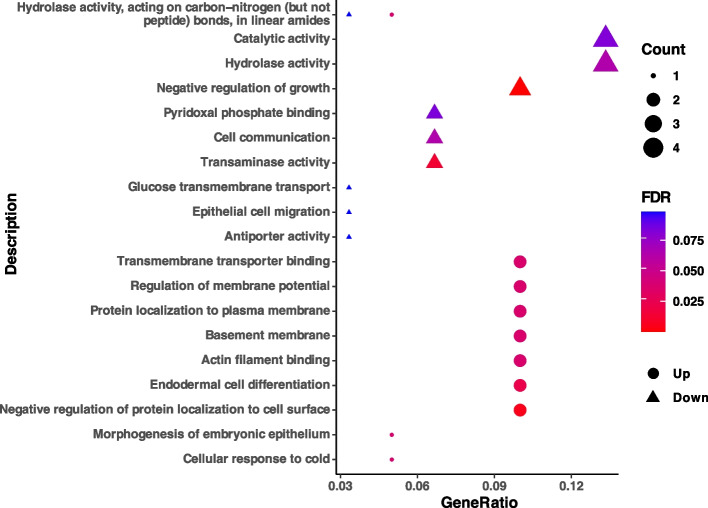
The GO enrichment of DEGs in the copper supplement group. Abbreviations: GO = gene ontology; DEGs = differentially expressed genes; FDR = false discovery rate

The results of GO assays revealed that DEGs of SCu were enriched in 19 terms compared with SCG, among which, 12 terms such as negative regulation of growth, transaminase activity, negative regulation of protein localization to cell surface, endodermal cell differentiation, regulation of membrane potential, basement membrane, transmembrane transporter binding, protein localization to plasma membrane, actin filament binding, hydrolase activity, acting on carbon–nitrogen (but not peptide) bonds, in linear amides, cellular response to cold, and morphogenesis of embryonic epithelium were significantly enriched (FDR < 0.05) (Fig. 5).

**Fig. 6:**
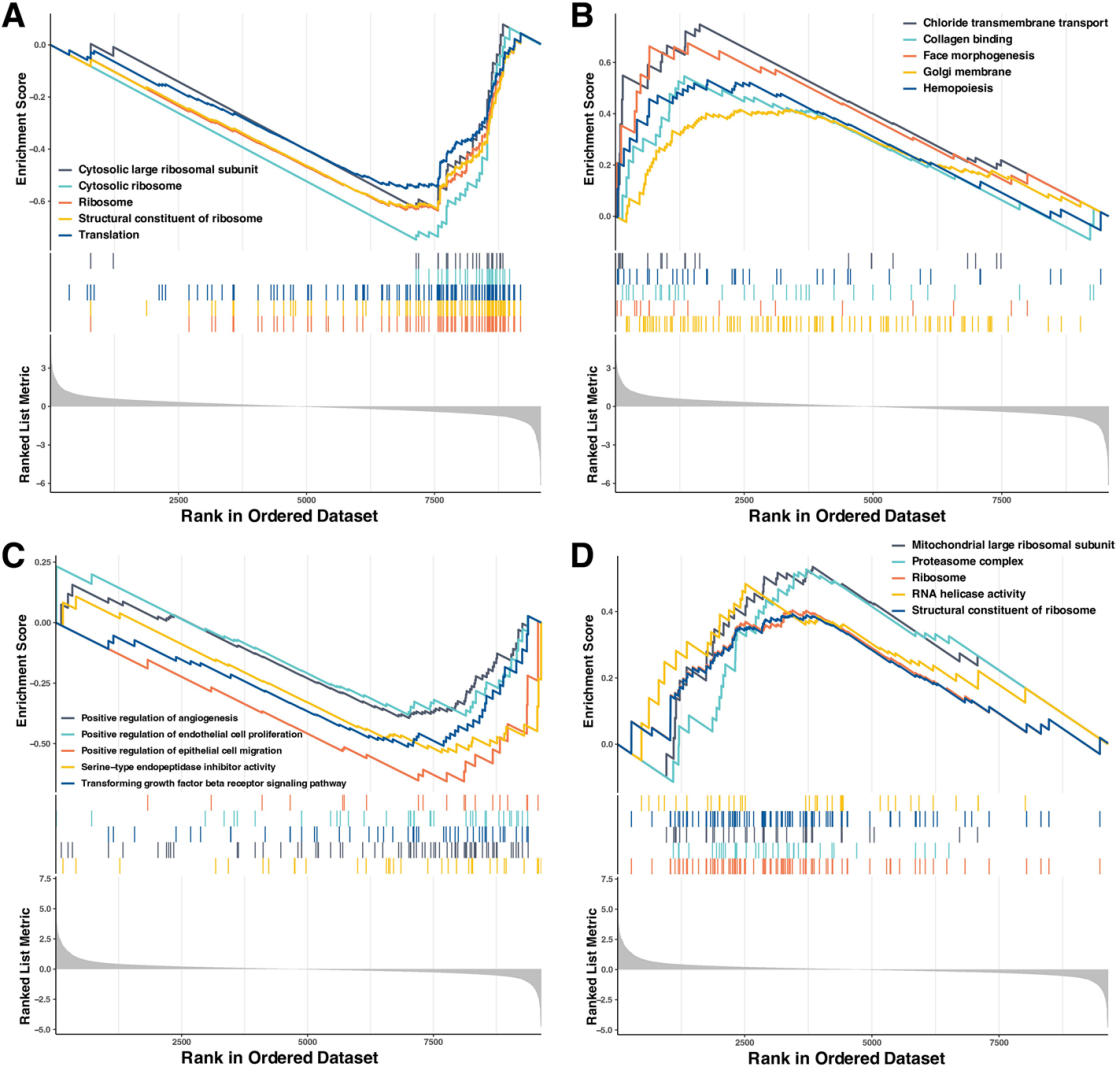
The Top 5 GSEA enrichment of GO terms. **A** Enriched in the control group of the copper deficient treatment period. **B** Enriched in the copper deficient group. **C** Enriched in the control group of the copper supplement treatment period. **D** Enriched in the copper supplement group. Abbreviations: GSEA = gene set enrichment analysis; GO = gene ontology

The results of GESA assays based on the GO enrichment data set were shown in Fig. 6. The top 5 significantly enriched terms sorted by normalized enrichment score (NES) in LCG were ribosome (NES, -3.41), structural constituent of ribosome (NES, -3.32), cytosolic ribosome (NES, -3.15), translation (NES, -2.93) and cytosolic large ribosomal subunit (NES, -2.79) (FDR < 0.25) (Fig. 6A). The top 5 significant terms of LCu were Golgi membrane (NES, 2.06), face morphogenesis (NES, 2.06), collagen binding (NES, 2.06), hemopoiesis (NES, 2.05) and chloride transmembrane transport (NES, 2.04) (FDR < 0.25) (Fig. 6B). The top 5 significant terms of SCG were serine-type endopeptidase inhibitor activity (NES, -2.20), positive regulation of angiogenesis (NES, -2.19), transforming growth factor beta receptor signaling pathway (NES, -2.11), positive regulation of endothelial cell proliferation (NES, -2.10) and positive regulation of epithelial cell migration (NES, -2.06) (FDR < 0.25) (Fig. 6C). The top 5 significant terms of SCu were ribosome (NES, 2.05), proteasome complex (NES, 2.03), mitochondrial large ribosomal subunit (NES, 2.01), structural constituent of ribosome (NES, 2.01) and RNA helicase activity (NES, 1.93) (FDR < 0.25) (Fig. 6D).

#### Screening of critical genes

**Fig. 7:**
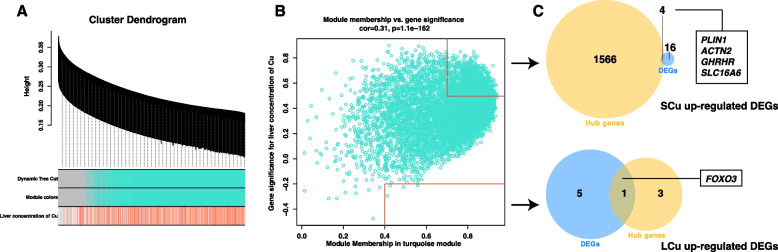
Screening of the candidate critical genes by WGCNA method. **A** Network dendrogram from co-expression topological overlap. Color bars show the correlation of gene expression with liver concentration of Cu. **B** The scatter plot of the association between the turquoise module and gene importance. The genes within the red box are identified as hub genes. **C** The Venn diagram of overlapping genes between DEGs and the hub genes as candidate critical genes. Abbreviations: WGCNA = the weighted gene co-expression network analysis; DEGs = differentially expressed genes; LCu = the low-Cu feeding group; SCG = the control group for the high-Cu feeding period

WGCNA analysis constructed a turquoise module, so the correlation data of liver Cu concentration and the turquoise module were selected to screen hub genes (Fig. 7A). Genes with module membership (MM) more than 0.70 and gene significance (GS) more than 0.50 were identified as hub genes of SCu up-regulated DEGs and LCu down-regulated DEGs, and a total of 1570 hub genes were screened. Genes with MM more than 0.40 and GS less than -0.20 were identified as hub genes of LCu up-regulated DEGs and SCu down-regulated DEGs, and a total of 4 hub genes were screened (Fig. 7B). The overlapping genes between DEGs and the hub genes were identified as candidate critical genes. One candidate critical gene was screened in the LCu up-regulated DEGs and 4 candidate critical genes were screened in the SCu up-regulated DEGs (Fig. 7C). There was no screening for candidate critical genes of LCu down-regulated DEGs and SCu down-regulated DEGs.

**Fig. 8:**
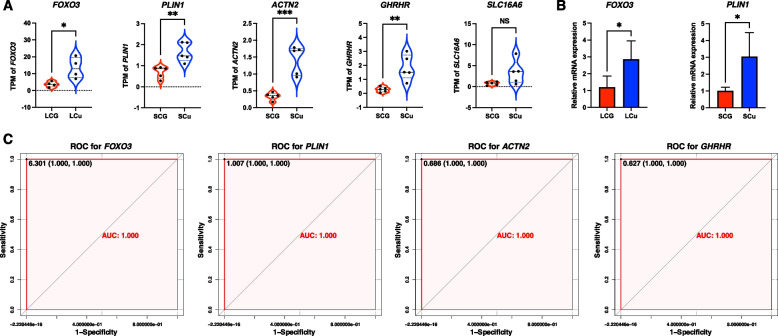
The validation of the critical genes. **A** The gene TPM counts of candidate critical genes. **B** The relative mRNA expression levels of *FOXO3* and *PLIN1* in the liver by qRT-PCR. **C** The ROC validation for critical genes. NS *p* > 0.05, * *p* < 0.05, ** *p* < 0.01, *** *p* < 0.001. Abbreviations: LCu = the low-Cu feeding group; LCG = the control group for the low-Cu feeding period; SCu = the high-Cu supplement group; SCG = the control group for the high-Cu feeding period; TPM = transcripts per kilobase million reads; qRT-PCR = quantitative real-time polymerase chain reaction; ROC = the receiver operating characteristic curve

Figure 8A showed the gene expression of candidate critical genes. Since gene *SLC16A6* (solute carrier family 16 member 6) expression of SCu was not significantly upregulated compared with SCG, it could not be considered as the critical gene (*p* > 0.05). *FOXO3* (forkhead box O3) and *PLIN1* (perilipin 1) two candidate critical genes were randomly chosen to validate the gene expression data. The mRNA expression level of *FOXO3* in the LCu liver and the mRNA expression level of *PLIN1* in the SCu liver were significantly increased by qRT-PCR verification (*p* < 0.05) (Fig. 8B). The area under curve (AUC) values of the ROC models for *FOXO3*, *PLIN1*, *ACTN2* (actinin alpha 2) and *GHRHR* (growth hormone releasing hormone receptor) were 1, which indicated that *FOXO3* can be considered a critical gene for LCu and *PLIN1*, *ACTN2* and *GHRHR* can be considered as critical genes for SCu (Fig. 8C).

### Metabolome analysis

**Fig. 9:**
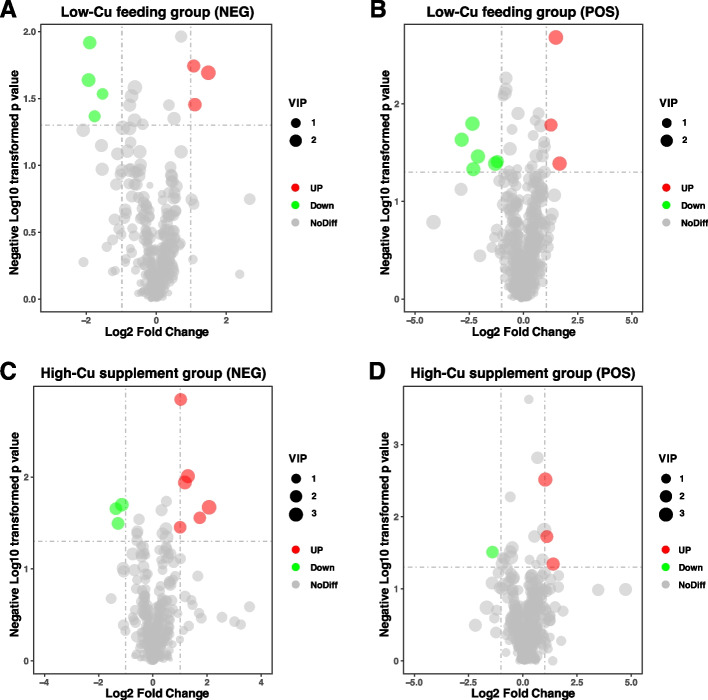
DEMs identified between different treatment groups. **A** Volcano Plot of DEMs in LCu of NEG; **B** Volcano Plot of DEMs in LCu of POS; **C** Volcano Plot of DEMs in SCu of NEG; **D** Volcano Plot of DEMs in SCu of POS. Abbreviations: LCu = the low-Cu feeding group; SCG = the control group for the high-Cu feeding period; NEG = negative ion mode; POS = positive ion mode; DEMs = significantly accumulated metabolites

To further explore how dietary Cu deficiency and supplementation cause liver metabolite changes, liver untargeted metabolomics were applied. The Partial Least Squares Discriminant Analysis (PLS-DA) revealed differences between LCu and LCG, SCu and SCG (Figure S3). As shown in Fig. 9A and B, a total of 7 DEMs were identified in LCu of negative ion mode (NEG), including 3 up-regulated and 4 down-regulated DEMs, 9 DEMs were identified in LCu of positive ion mode (POS), including 3 up-regulated and 6 down-regulated DEMs. These DEMs were mainly classified into steroids and steroid derivatives and fatty acyls. In the SCu (NEG), 9 DEMs were identified, including 6 up-regulated and 3 down-regulated DEMs, 4 DEMs were identified in SCu (POS), including 3 up-regulated and 1 down-regulated DEMs (Fig. 9C, D). The DEMs of SCu were mainly classified into fatty acyls and carboxylic acids and derivatives. The up-regulation and down-regulation of DEMs information were shown in Table S7 (*p* < 0.05).

**Fig. 10:**
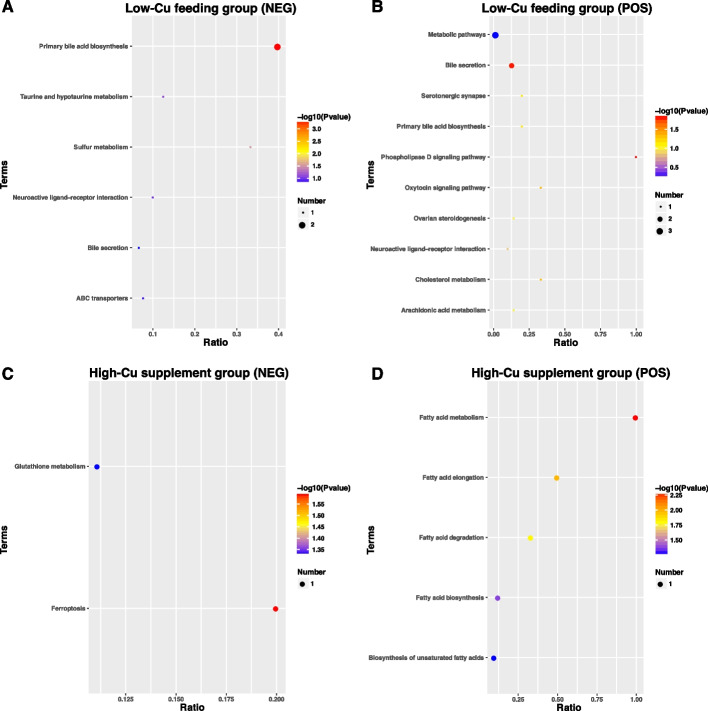
The KEGG pathways enrichment of DEMs in the LCu of NEG (**A**), LCu of POS (**B**), SCu of NEG (**C**) and SCu of POS (**D**). Abbreviations: KEGG = the Kyoto Encyclopedia of Genes and Genomes; DEMs = significantly accumulated metabolites; LCu = the low-Cu feeding group; SCG = the control group for the high-Cu feeding period; NEG = negative ion mode; POS = positive ion mode

KEGG analysis of the DEMs revealed that 6 pathways were enriched in DEMs (NEG) of LCu, among which the pathway of primary bile acid biosynthesis and sulfur metabolism were significantly enriched (Fig. 10A), 10 pathways were enriched in DEMs (POS) of LCu, among which the pathway of phospholipase D signaling pathway, bile secretion, oxytocin signaling pathway and cholesterol metabolism were significantly enriched (Fig. 10B), the pathway of ferroptosis and glutathione metabolism were significantly enriched in DEMs (NEG) of SCu (Fig. 10C), 5 pathways were enriched in DEMs (POS) of SCu, among which the pathway of fatty acid metabolism, fatty acid elongation, fatty acid degradation and fatty acid biosynthesis were significantly enriched (Fig. 10D) (*p* < 0.05). The main secondary classifications of these KEGG pathways are lipid metabolism and digestive system.

### Integrative analyses of transcriptome and metabolome

**Fig. 11:**
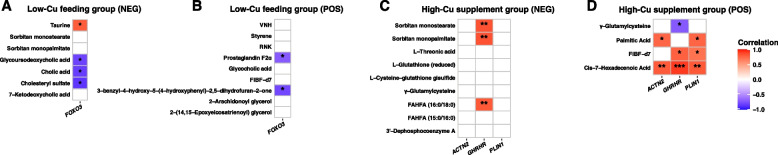
The correlation analysis of critical genes and DEMs in the LCu of NEG (**A**), LCu of POS (**B**), SCu of NEG (**C**) and SCu of POS (**D**). * *p* < 0.05, ** *p* < 0.01, *** *p* < 0.001. Abbreviations: DEMs = significantly accumulated metabolites; LCu = the low-Cu feeding group; SCG = the control group for the high-Cu feeding period; NEG = negative ion mode; POS = positive ion mode

Correlation analysis of LCu critical gene and LCu DEMs (NEG) revealed that the *FOXO3* was significantly positively correlated with taurine and significantly negatively correlated with cholesteryl sulfate, glycoursodeoxycholic acid, and cholic acid (*p* < 0.05) (Fig. 11A). *FOXO3* was significantly negatively associated with prostaglandin f2α and 3-benzyl-4-hydroxy-5-(4-hydroxyphenyl)-2,5-dihydrofuran-2-one in the LCu (POS) (*p* < 0.05) (Fig. 11B).

Correlation analysis of SCu critical genes and SCu DEMs (NEG) revealed that *GHRHR* was significantly positively associated with FAHFA (16:0/18:0), sorbitan monopalmitate and sorbitan monostearate (*p* < 0.05) (Fig. 11C). In the SCu (POS), *PLIN1* was significantly positively associated with cis-7-hexadecenoic acid, palmitic acid and FIBF-d7, *ACTN2* was significantly positively associated with cis-7-hexadecenoic acid and palmitic acid, *GHRHR* was significantly positively correlated with cis-7-hexadecenoic acid, FIBF-d7 and significantly negatively correlated with γ-Glutamylcysteine (*p* < 0.05) (Fig. 11D).

## Discussion

Trace mineral elements are essential nutrients for the growth and development of ruminants. Cu is present in various proteins and enzymes, and maintaining an appropriate concentration of Cu is essential for effectively carrying out its physiological, catalytic, and regulatory functions [22]. Dietary Cu deficiency and supplementation did not significantly alter liver weight, which indicated that Cu status in grazing sheep does not affect liver weight variation. The liver serves as the primary storage organ for Cu. In cases of Cu deficiency among cattle, the liver's Cu concentration will decrease initially [23]. Due to the liver playing a central role in metabolism, it can serve as a Cu source to maintain Cu homeostasis in ruminants when Cu intake is insufficient [24]. In this experiment, dietary Cu deficiency and supplementation of grazing sheep led to significant reductions and elevations in the liver concentration of Cu, respectively, which was consistent with our previous findings in serum [11]. These results indicated that the liver of grazing sheep was highly responsive to changes in dietary Cu concentration. The average concentration of Cu in the liver of LCu grazing sheep was found to be 0.0049 mg/g, which was in Cu deficiency status [25]. However, supplementation of Cu increased its liver concentration to 0.062 mg/g, suggesting that the Cu status of grazing sheep was adequate [26]. It is worth noting that Cu–Zn antagonism in the rumen of ruminants can lead to reduced Zn absorption in the rumen and intestine, resulting in a significant reduction in liver Zn concentration in Cu supplement treatment grazing sheep [27]. Meanwhile, due to the multi-nutrient salts containing Ca and Se in the diet can significantly elevate the liver concentration of Ca in LCu and the liver concentration of Se in SCu, respectively.

Transcriptome analysis can reveal a series of genes that exhibit differential expression in response to dietary Cu deficiency and supplementation in grazing sheep. As a data reduction method and an unsupervised classification method, the WGCNA was a hybrid. Modules were reduced to a handful of easily interpreted gene responses [17]. In this experiment, the phenotype information of liver Cu concentration was co-analyzed with WGCNA to find the hub genes. Finally, *FOXO3* was screened as the critical gene by WGCNA and DESeq2 analysis in LCu. *FOXO3* is a member of the FOXO (forkhead box class O) family that plays an important role in cell cycle control, apoptosis, neural and hematopoietic cell differentiation and DNA repair [28]. The FOXO family includes *FOXO1*, *FOXO3*, *FOXO4* and *FOXO6*. *FOXO3* is associated with many age-related diseases, including cancer, cardiovascular disease, disc degeneration, and neurodegenerative diseases [29]. The research on the regulation of liver regeneration revealed that *FOXO3* could limit liver regeneration by inhibiting hepatocyte proliferation [30]. Notably, *FOXO3* was found to be enriched in the GO term of DNA damage response that exhibited significant enrichment in LCu. Additionally, dietary Cu deficiency was observed to significantly enrich ribosome-related GO terms in LCG during GSEA analysis. Ribosomes play a fundamental role in translating genomes and synthesizing proteins [31]. These findings suggest that dietary Cu deficiency may result in DNA damage, impaired liver regeneration, and compromised ribosome function in grazing sheep.

*PLIN1*, *ACTN2*, and *GHRHR* were screened as up-regulated critical genes by WGCNA and DESeq2 analysis in SCu. Lipid droplets (LDs) were universal cellular organelles that were major stores of energy and lipids in eukaryotic cells [32]. Periilipins (PLINs) were a family of structural proteins associated with the surface of LDs [33]. PLIN1 was the most abundant protein around LDs which played a key role in lipid homeostasis [32]. Integrative analysis of transcript profiles and fatty acid profiles showed that *PLIN1* accelerates intramuscular fat deposition by positively regulating saturated and monounsaturated fatty acid metabolism in Tan sheep [34]. It has also been found that some sterol biosynthetic enzymes were downregulated in adipose tissue of *PLIN1* deficient mice, which suggested that *PLIN1* deficiency may affect the sterol biosynthetic pathway [35]. In contrast, overexpression of *PLIN1* promoted lipid accumulation in chicken preadipocytes [32]. Interestingly, *PLIN1* was found to be enriched in the GO terms of cellular response to cold that exhibited significantly enriched in SCu. *ACTN2* encoded alpha-actinin-2, which was expressed in cardiac and skeletal muscle, and the protein contributed to the stabilization of the sarcomere [36]. The findings of this study align with the significantly enriched GO terms in SCu associated with *ACTN2*. These terms include regulation of membrane potential, transmembrane transporter binding, protein localization to the plasma membrane, and actin filament binding. GHRHR was a G protein-coupled receptor. Growth hormone was an important pituitary hormone that served as a key regulator of growth, metabolism, and immune regulation. Its synthesis and release were mainly regulated by GHRHR-mediated intracellular signals [37]. It was also found by GSEA analysis that dietary Cu supplementation led to a significant enrichment of ribosome-related GO terms in SCu. These findings suggest that dietary Cu supplementation could potentially enhance ribosome function, promote lipid accumulation, and stimulate growth and metabolism in grazing sheep.

Metabolomic analysis was utilized to identify the DEMs resulting from dietary deficiency and supplementation of Cu in grazing sheep. A total of 16 DEMs were identified in the LCu and 13 DEMs were identified in the SCu. Among them, most DEMs induced in response to Cu treatment were classified as steroids and steroid derivatives, and fatty acyls, which all belong to the lipids and lipid-like molecules superclass. KEGG pathway analysis detected that these DEMs mainly belonged to the lipid metabolism and digestive system. The results of the KEGG pathway analysis revealed that dietary Cu deficiency may decrease hepatic bile secretion and cholesterol metabolism, while dietary Cu supplementation may elevate hepatic fatty acid synthesis and metabolism in grazing sheep.

Through integrative analysis of the transcriptome and metabolome, *FOXO3* was found to have a significant and negative correlation with cholesteryl sulfate, glycoursodeoxycholic acid, cholic acid and prostaglandin f2α in the LCu. Among them, cholesterol sulfate was an endogenous regulator of cholesterol synthesis that inhibited glutamate-induced cell death in HT-2 cells and reduced reactive oxygen species production [38]. Glycoursodeoxycholic acid can alleviate endoplasmic reticulum stress and hepatic steatosis in mice caused by the high-fat diet [39]. Cholic acid was an important bile acid that was usually used to enhance the absorption of cholesterol [40]. In addition, a study conducted on mice revealed that a Cu-deficient diet resulted in an 80% reduction in the mRNA abundance of cholesterol 7α-hydroxylase, a critical enzyme involved in cholic acid synthesis [41, 42]. The reduction in cholic acid abundance could be attributed to this mechanism. Prostaglandin f2α was a bioactive lipid metabolite of arachidonic acid, which exerted physiological function by binding to its receptors. In animal experiments, feeding high fat diets caused the liver prostaglandin f2α product significantly increased [43]. The correlation result indicated that the up-regulation gene *FOXO3* can negatively regulate cholesteryl sulfate, glycoursodeoxycholic acid, cholic acid and prostaglandin f2α, which led to a significant decrease in the abundance of the four metabolites. This demonstrated that dietary Cu deficiency may reduce liver lipid metabolism in grazing sheep. Lipids were small molecules with multiple chemical structures that played a crucial role in almost all aspects of cellular function [44].

In the SCu, *GHRHR* was significantly and positively correlated with FAHFA (16:0/18:0). FAHFA is an endogenous lipid that promotes glucose transport and secreted insulin, which had a preventive effect on diabetes [45]. *PLIN1* and *ACTN2* were found to have a significant positive correlation with palmitic acid. Lipidomics analysis has revealed that palmitic acid impaired the development of hepatocellular carcinoma by regulating cell membrane fluidity and glucose metabolism [44]. Furthermore, research has demonstrated that adding Cu to the diet leads to higher concentrations of fatty acids in calf plasma [46]. This effect may be due to the crucial role of Cu in the activity of enzymes involved in fatty acid synthesis and metabolism. The correlation analysis showed that the up-regulation gene *PLIN1*, *ACTN2* and *GHRHR* can positively regulate the above up-regulated metabolites of SCu. This suggested that dietary supplementation of Cu may elevate liver lipid metabolism in grazing sheep.

However, this experiment did not identify any complementary DEMs under Cu-deficient and Cu-supplemented conditions. This could be attributed to the regulation of metabolite abundance by multiple factors, including other nutrients, gene expression, and competition within metabolic pathways. Even with adequate Cu supplementation, if other factors restrict metabolite synthesis or enhance its degradation, the increase in abundance may not be significant. Additionally, changes in metabolite abundance may exhibit a time delay. It may take some time for the activity of Cu-dependent enzymes to sufficiently increase after Cu supplementation, resulting in a notable impact on metabolite abundance. Furthermore, further analysis is required to comprehend the mechanism of gene regulation in relation to metabolite alterations.

In addition, this study still has several limitations. Firstly, the feeding experiments could be better designed for 40, 80, or 120 days to obtain more rational days of supplementation for grazing sheep. Secondly, lipidomics should be further measured to explore the precise effects of dietary Cu status on lipid metabolism. Thirdly, the experiment results may be influenced by the animal species and the variability of individual animals. Therefore, conducting further studies involving different sheep breeds is necessary to validate and expand upon our findings, ultimately leading to a more comprehensive understanding of this study.

## Conclusions

This study explored the effect of dietary Cu deficiency and supplementation on the liver weight, concentration of mineral elements, transcriptomic and metabolomic in grazing sheep. The findings revealed that dietary Cu treatment did not affect liver weight, and the liver concentration of Cu was susceptible to variation due to dietary Cu. *FOXO3*, *PLIN1*, *ACTN2* and *GHRHR* were identified as critical genes using WGCNA and random qRT-PCR validation that could serve as biomarkers. Combining these critical genes with gene functional enrichment analysis, it was observed that dietary Cu deficiency may impair liver regeneration and compromise ribosomal function. Conversely, dietary Cu supplementation may enhance ribosomal function, promote lipid accumulation, and stimulate growth and metabolism in grazing sheep. Integrative analysis of the transcriptome and metabolome indicated that dietary Cu deficiency may reduce lipid metabolism while Cu supplementation may elevate it in grazing sheep. These findings provide new insights into the effects of dietary Cu deficiency and supplementation on sheep liver and provide the basis for further exploration of the mechanisms of critical genes regulating lipid metabolism in grazing ruminants.

### Supplementary Information

**Supplementary Material 1. **

## Data Availability

The sequencing data will be deposited in the public archive of NCBI under the BioProject ID PRJNA1024932.

## Abbreviations

Cu: Copper
Fe: Iron
NRC: National Research Council
LCu: The low-Cu feeding group
LCG: The control group for the low-Cu feeding period
SCu: The high-Cu supplement group
SCG: The control group for the high-Cu feeding period
Ca: Calcium
P: Phosphorus
S: Sulphur
K: Potassium
Co: Cobalt
Zn: Zinc
Mn: Manganese
Se: Selenium
Mo: Molybdenum
DEGs: Differentially expressed genes
FDR: False discovery rate
WGCNA: The weighted gene co-expression network analysis
ME: Module eigengene
ROC: The receiver operating characteristic curve
qRT-PCR: Quantitative real-time polymerase chain reaction
GO: Gene ontology
GSEA: Gene set enrichment analysis
VIP: Variable importance in projection
DEMs: Significantly accumulated metabolites
KEGG: The kyoto encyclopedia of genes and genomes
SE: Standard error
NES: Normalized enrichment score
MM: Module membership
GS: Gene significance
AUC: The area under curve
PLS-DA: The partial least squares discriminant analysis
NEG: Negative ion mode
POS: Positive ion mode
