# Supplementary material for: Transcriptomics and metabolomics analysis reveal the dietary copper deficiency and supplementation effects of liver gene expression and metabolite change in grazing sheep
Source: BMC Genomics. 2024 Feb 27;25:220. doi: 10.1186/s12864-024-10134-3 (PMC10900733; doi:10.1186/s12864-024-10134-3)
Supplement: Supplementary file 1 — Supplementary Material 1. [file 12864_2024_10134_MOESM1_ESM.docx]

**Transcriptomics and metabolomics analysis reveal the dietary copper deficiency and supplementation effects of liver gene expression and metabolite change in grazing Sheep**

Xiwei Jin, Lingbo Meng, Zhi Qi, Lan Mi^*^

**Supplementary material**

**Fig. S1.** The result of FastQC quality control. The sequence quality of LCu and LCG (A), SCu and SCG (C). The per sequence quality scores of LCu and LCG (B), SCu and SCG (D). Abbreviations: LCu = the low-Cu feeding group; LCG = the control group for the low-Cu feeding period; SCu = the high-Cu supplement group; SCG = the control group for the high-Cu feeding period.

**
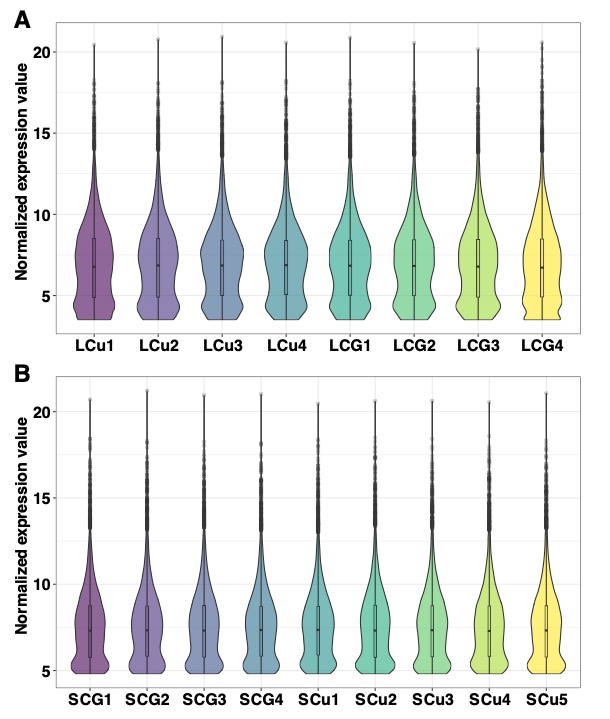
**

**Fig. S2.** The normalized expression value of LCu and LCG (A), SCu and SCG (B) by DESeq2. Abbreviations: LCu = the low-Cu feeding group; LCG = the control group for the low-Cu feeding period; SCu = the high-Cu supplement group; SCG = the control group for the high-Cu feeding period.

**Fig. S3.** The partial least squares discriminant analysis of LCu and LCG (A, B), SCu and SCG (C, D). R2Y values higher than Q2Y values indicate that the model quality is reliable. Abbreviations: LCu = the low-Cu feeding group; LCG = the control group for the low-Cu feeding period; SCu = the high-Cu supplement group; SCG = the control group for the high-Cu feeding period; NEG = negative ion mode; POS = positive ion mode.

**Table S1 Formulation of the multi-nutrient salts for grazing Wu Ranke sheep**

| **Group** | **Types** | **Amounts** | **Types** | **Amounts** | **Types** | **Amounts** |
| --- | --- | --- | --- | --- | --- | --- |
| **Control group** | KCl | 11.5 g/d | MgO | 1.7 g/d | Ca(IO_3_)_2_ | 1.4 mg/d |
|  | CaCO_3_ | 10 g/d | FeSO_4_ | 200 mg/d | CoSO_4_ | 1.2 mg/d |
|  | Na_2_SO_4_ | 9 g/d | ZnSO_4_ | 132 mg/d | Na_2_SeO_3_ | 1 mg/d |
|  | NH_4_H_2_PO_4_ | 7.4 g/d | MnSO_4_ | 92 mg/d |  |  |
|  | NaCl | 5 g/d | CuSO_4_ | 36 mg/d |  |  |
| **Low-Cu feeding group** | KCl | 11.5 g/d | MgO | 1.7 g/d | Ca(IO_3_)_2_ | 1.4 mg/d |
|  | CaCO_3_ | 10 g/d | FeSO_4_ | 200 mg/d | CoSO_4_ | 1.2 mg/d |
|  | Na_2_SO_4_ | 9 g/d | ZnSO_4_ | 132 mg/d | Na_2_SeO_3_ | 1 mg/d |
|  | NH_4_H_2_PO_4_ | 7.4 g/d | MnSO_4_ | 92 mg/d |  |  |
|  | NaCl | 5 g/d | CuSO_4_ | 0 mg/d |  |  |
| **High-Cu supplement group** | KCl | 11.5 g/d | MgO | 1.7 g/d | Ca(IO_3_)_2_ | 1.4 mg/d |
|  | CaCO_3_ | 10 g/d | FeSO_4_ | 200 mg/d | CoSO_4_ | 1.2 mg/d |
|  | Na_2_SO_4_ | 9 g/d | ZnSO_4_ | 132 mg/d | Na_2_SeO_3_ | 1 mg/d |
|  | NH_4_H_2_PO_4_ | 7.4 g/d | MnSO_4_ | 92 mg/d |  |  |
|  | NaCl | 5 g/d | CuSO_4_ | 108 mg/d |  |  |

**Table S2 Nutrient content in oats and mixed forages**

| **Items** | **Oat** | **Natural forage** |
| --- | --- | --- |
| P (mg/g) | 2.40 | 0.65 |
| S (mg/g) | 1.39 | 1.09 |
| K (mg/g) | 5.05 | 11.83 |
| Ca (mg/g) | 0.52 | 7.43 |
| Mn (ug/g) | 41.00 | 54.00 |
| Fe (ug/g) | 112.00 | 566.00 |
| Co (ug/g) | - | - |
| Cu (ug/g) | 3.50 | 4.50 |
| Zn (ug/g) | 21.00 | 17.00 |
| Se (ug/g) | 0.090 | - |
| Crude Protein % | 10.36 | 8.25 |
| Acid Detergent Fiber % | 5.27 | 30.15 |
| Neutral Detergent Fiber % | 11.72 | 65.07 |

**Table S3 The digestion procedure of the microwave digester**

| **steps** | **Control temperature (℃)** | **Heating time (min)** | **Constant temperature time (min)** |
| --- | --- | --- | --- |
| 1 | 120 | 5 | 5 |
| 2 | 150 | 5 | 10 |
| 3 | 190 | 5 | 20 |

**Table S4 The primers sequences**

| **Gene name** | **Forward (5’ to 3’)** | **Reserve (5’ to 3’)** |
| --- | --- | --- |
| *FOXO3* | CTTCAGCAGCGCGGTATTTG | GGTCCTGGAGTGTCTGGTTG |
| *PLIN1* | ACCGTCTCAGTCGTGATGTG | ACGTAGTGCACCACTGTGTC |
| *GAPDH* | CGTGTCCGTTGTGGATCTGA | TGAAGTCGCAGGAGACAACC |

**Table S5 The chromatographic and** **mass spectrometric conditions of UHPLC-MS/MS**

|  | **Type** | **Parameters** |
| --- | --- | --- |
| **The**  **chromatographic**  **conditions** | Chromatographic column | Hypesil Goldcolumn (C18) |
|  | Column temperature | 40 °C |
|  | Flow rate | 0.2 mL/min |
|  | Positive polarity mode | Eluent A (0.1% FA in Water) |
|  |  | Eluent B (Methanol) |
|  | Negative polarity mode | Eluent A (5 mM ammonium acetate, pH 9.0) |
|  |  | Eluent B (Methanol) |
| **The**  **mass**  **spectrometric**  **conditions** | Spray Voltage | 3.2 kV |
|  | Sheath gas flow rate | 40 arb |
|  | Aux Gasflow rate | 10 arb |
|  | Capillary Temp | 320 °C |
|  | Polarity | Positive; negative |

**Table S6 The differentially expressed genes identified in Cu treatment group*^a^***

| **Group** | **Genes** | **Log_2_FC** | **FDR** | **Up/Down** |
| --- | --- | --- | --- | --- |
| LCu | *HIPK3* | 1.328 | 0.000 | Up |
|  | *OCIAD2* | 3.629 | 0.001 | Up |
|  | *TPSB2* | 3.344 | 0.007 | Up |
|  | *A2M* | 1.2 | 0.009 | Up |
|  | *AGL* | 1.965 | 0.012 | Up |
|  | *FOXO3* | 1.604 | 0.021 | Up |
| SCu | *LPIN1* | -2.415 | 0.000 | Down |
|  | *VNN1* | 1.883 | 0.000 | Up |
|  | *ARG2* | -4.509 | 0.000 | Down |
|  | *MT2* | -4.797 | 0.000 | Down |
|  | *ACACB* | 1.804 | 0.000 | Up |
|  | *KHK* | 1.231 | 0.000 | Up |
|  | *RETREG1* | -1.984 | 0.000 | Down |
|  | *AMDHD1* | -1.503 | 0.000 | Down |
|  | *PLBD1* | -1.653 | 0.000 | Down |
|  | *ACTN2* | 1.902 | 0.000 | Up |
|  | *GSTA4* | -2.295 | 0.000 | Down |
|  | *MT1A* | -4.143 | 0.000 | Down |
|  | *EGFL8* | -1.902 | 0.001 | Down |
|  | *GPT2* | -1.389 | 0.001 | Down |
|  | *LAMA5* | 1.092 | 0.002 | Up |
|  | *GHRHR* | 2.735 | 0.002 | Up |
|  | *SERPINA6* | -2.003 | 0.002 | Down |
|  | *ACMSD* | -1.69 | 0.002 | Down |
|  | *TAF6L* | 1.082 | 0.002 | Up |
|  | *ENPP5* | -1.028 | 0.002 | Down |
|  | *SCD* | -1.817 | 0.003 | Down |
|  | *MORC4* | -1.313 | 0.003 | Down |
|  | *ANKEF1* | 1.543 | 0.003 | Up |
|  | *TMOD4* | 1.24 | 0.003 | Up |
|  | *SLC2A1* | -1.15 | 0.004 | Down |
|  | *MYOZ1* | -2.344 | 0.005 | Down |
|  | *HAL* | -1.146 | 0.006 | Down |
|  | *OAT* | -1.433 | 0.006 | Down |
|  | *TM7SF2* | -1.403 | 0.007 | Down |
|  | *RCN3* | 1.241 | 0.007 | Up |
|  | *GCA* | -1.871 | 0.008 | Down |
|  | *CDK3* | 1.25 | 0.012 | Up |
|  | *PAXBP1* | 1.02 | 0.012 | Up |
|  | *SLC17A2* | 1.089 | 0.012 | Up |
|  | *SLC8A3* | -1.598 | 0.013 | Down |
|  | *ACOT12* | 1.51 | 0.017 | Up |
|  | *SLC16A6* | 2.472 | 0.020 | Up |
|  | *NEDD4L* | 1.072 | 0.023 | Up |
|  | *IRS2* | -1.047 | 0.023 | Down |
|  | *FREM1* | -1.514 | 0.024 | Down |
|  | *MERTK* | -1.105 | 0.028 | Down |
|  | *SLC7A2* | -1.242 | 0.031 | Down |
|  | *ACER2* | -1.276 | 0.031 | Down |
|  | *LAMB3* | 1.188 | 0.031 | Up |
|  | *PLIN1* | 1.308 | 0.032 | Up |
|  | *ITGA7* | 1.023 | 0.036 | Up |
|  | *RNF125* | -1.188 | 0.040 | Down |
|  | *AASS* | -1.11 | 0.042 | Down |
|  | *MT1C* | -3.123 | 0.045 | Down |
|  | *FBXO44* | 1.6 | 0.046 | Up |

*^a^* LCu = the low-Cu feeding group; SCu = the high-Cu supplement group; Log_2_FC: Log_2_Fold change.

**Table S7** **The significantly accumulated metabolites identified in Cu treatment group*^a^***

| **Group** | **Metabolites** | **VIP** | **Log_2_FC** | ***p* value** | **Up/Down** | **Class (HMDB)** |
| --- | --- | --- | --- | --- | --- | --- |
| **LCu (NEG)** | Cholesteryl sulfate | 2.233 | -1.904 | 0.012 | Down | Steroids and steroid derivatives |
|  | Sorbitan monopalmitate | 2.172 | 1.069 | 0.018 | Up | Fatty Acyls |
|  | Taurine | 2.892 | 1.487 | 0.020 | Up | Organic sulfonic acids and derivatives |
|  | Glycoursodeoxycholic acid | 2.442 | -1.936 | 0.023 | Down | Steroids and steroid derivatives |
|  | Cholic acid | 1.511 | -1.537 | 0.029 | Down | Steroids and steroid derivatives |
|  | Sorbitan monostearate | 2.330 | 1.104 | 0.035 | Up | Fatty Acyls |
|  | 7-Ketodeoxycholic acid | 1.668 | -1.764 | 0.043 | Down | Steroids and steroid derivatives |
| **LCu (POS)** | 2-Arachidonoyl glycerol | 2.921 | 1.495 | 0.002 | Up | -- |
|  | Glycocholic acid | 2.771 | -2.354 | 0.016 | Down | Steroids and steroid derivatives |
|  | FIBF-d7 | 2.182 | 1.268 | 0.016 | Up | -- |
|  | RNK | 2.675 | -2.851 | 0.023 | Down | -- |
|  | Styrene | 2.721 | -2.100 | 0.035 | Down | Benzene and substituted derivatives |
|  | 3-benzyl-4-hydroxy-5-(4-hydroxyphenyl)-2,5-dihydrofuran-2-one | 2.121 | -1.206 | 0.039 | Down | -- |
|  | 2-(14,15-Epoxyeicosatrienoyl) glycerol | 2.757 | 1.665 | 0.041 | Up | Glycerolipids |
|  | Prostaglandin F2α | 2.928 | -1.297 | 0.041 | Down | Fatty Acyls |
|  | VNH | 2.604 | -2.313 | 0.047 | Down | -- |
| **SCu (NEG)** | FAHFA (16:0/18:0) | 2.311 | 1.026 | 0.001 | Up | -- |
|  | L-Threonic acid | 3.029 | 1.293 | 0.010 | Up | -- |
|  | Sorbitan monopalmitate | 2.892 | 1.181 | 0.011 | Up | Fatty Acyls |
|  | γ-Glutamylcysteine | 2.812 | -1.145 | 0.020 | Down | Carboxylic acids and derivatives |
|  | L-Cysteine-glutathione gisulfide | 3.428 | 2.074 | 0.021 | Up | -- |
|  | L-Glutathione (reduced) | 2.318 | -1.365 | 0.022 | Down | -- |
|  | FAHFA (15:0/16:0) | 2.228 | 1.731 | 0.028 | Up | -- |
|  | 3'-Dephosphocoenzyme A | 2.326 | -1.285 | 0.032 | Down | -- |
|  | Sorbitan monostearate | 2.298 | 1.006 | 0.035 | Up | Fatty Acyls |
| **SCu (POS)** | Cis-7-Hexadecenoic Acid | 3.022 | 1.037 | 0.003 | Up | -- |
|  | Palmitic Acid | 2.320 | 1.108 | 0.019 | Up | Fatty Acyls |
|  | γ-Glutamylcysteine | 2.160 | -1.400 | 0.031 | Down | -- |
|  | FIBF-d7 | 2.492 | 1.400 | 0.045 | Up | -- |

*^a^* LCu = the low-Cu feeding group; SCu = the high-Cu supplement group; NEG: negative ion mode; POS: positive ion mode; VIP: variable importance in projection; Log_2_FC**:** Log_2_Fold change.
